# Factors influencing place of delivery for women in Kenya: an analysis of the Kenya demographic and health survey, 2008/2009

**DOI:** 10.1186/1471-2393-13-40

**Published:** 2013-02-17

**Authors:** John Kitui, Sarah Lewis, Gail Davey

**Affiliations:** 1 Medical Doctor and Public Health practitioner, PO Box 4798, Eldoret, Kenya; 2 Faculty of Medicine & Health Sciences, University of Nottingham, Room C116 Clinical Sciences Building, Nottingham City Hospital, Hucknall Road, Nottingham, NG5 1PB, UK; 3 Brighton & Sussex Medical School, Rm 2.16, Brighton, BN1 9PX, UK

**Keywords:** Maternal and child health, Maternal mortality, Neonatal mortality, Still birth, Delivery

## Abstract

**Background:**

Maternal mortality in Kenya increased from 380/100000 live births to 530/100000 live births between 1990 and 2008. Skilled assistance during childbirth is central to reducing maternal mortality yet the proportion of deliveries taking place in health facilities where such assistance can reliably be provided has remained below 50% since the early 1990s. We use the 2008/2009 Kenya Demographic and Health Survey data to describe the factors that determine where women deliver in Kenya and to explore reasons given for home delivery.

**Methods:**

Data on place of delivery, reasons for home delivery, and a range of potential explanatory factors were collected by interviewer-led questionnaire on 3977 women and augmented with distance from the nearest health facility estimated using health facility Global Positioning System (GPS) co-ordinates. Predictors of whether the woman’s most recent delivery was in a health facility were explored in an exploratory risk factor analysis using multiple logistic regression. The main reasons given by the woman for home delivery were also examined.

**Results:**

Living in urban areas, being wealthy, more educated, using antenatal care services optimally and lower parity strongly predicted where women delivered, and so did region, ethnicity, and type of facilities used. Wealth and rural/urban residence were independently related. The effect of distance from a health facility was not significant after controlling for other variables. Women most commonly cited distance and/or lack of transport as reasons for not delivering in a health facility but over 60% gave other reasons including 20.5% who considered health facility delivery unnecessary, 18% who cited abrupt delivery as the main reason and 11% who cited high cost.

**Conclusion:**

Physical access to health facilities through distance and/or lack of transport, and economic considerations are important barriers for women to delivering in a health facility in Kenya. Some women do not perceive a need to deliver in a health facility and may value health facility delivery less with subsequent deliveries. Access to appropriate transport for mothers in labour and improving the experiences and outcomes for mothers using health facilities at childbirth augmented by health education may increase uptake of health facility delivery in Kenya.

## Background

Approximately 1000 women die each day worldwide from pregnancy related causes, 99% of them in developing countries and more than 50% in sub-Saharan Africa [1] with most deaths concentrated around the time of delivery. An estimated 2.65 million stillbirths occurred in 2008 worldwide [2] while 3 million new-borns do not survive the first month of life worldwide annually [3]. Skilled assistance during childbirth, readily accessible appropriate care in case of complications and effective postnatal care within the first 24 hours of delivery are strategies that can improve perinatal outcomes for mothers and babies [4-6]. A key strategy to reducing maternal and neonatal deaths is the ‘health-centre intrapartum care strategy’, where qualified skilled workers manage labour, effectively manage complications and are supported with effective referral systems for specialised care when needed, and an effective postnatal care package [4,7].

A significant proportion of mothers in developing countries still deliver at home unattended by skilled health workers [5,8]. In diverse contexts, individual factors including maternal age, parity, education and marital status, household factors including family size, household wealth, and community factors including socioeconomic status, community health infrastructure, region, rural/urban residence, available health facilities, and distance to health facilities determine place of delivery and these factors interact in diverse ways in each context to determine place of delivery [9-11]. Eijk et al. looked at antenatal care and delivery care among women in Western Kenya and demonstrated that older women, high parity, lower socioeconomic status, low education levels and more than an hour walking distance were associated with delivery outside health facilities[12]. Studying poor urban dwellers in Nairobi, Fosto et al. found from bivariate analyses that wealth, education, parity, place of residence were associated with place of delivery [13]. Ochako has previously demonstrated that these factors together with marital status and age at birth of last child determined use and timing of first Antenatal Care (ANC) visit and type of delivery [14]. There are also wide variations in the reasons women give for delivering at home between and within countries [8,15-17]. For Kenya, recent studies looking at the degree of effect of such factors are lacking.

In Kenya, maternal mortality rate has not reduced over recent years, and may even have increased from an estimated 380/100000 live births in 1990 to 530/100000 live births in 2008 [1]. Although a number of factors may have contributed to this, including improved identification of maternal deaths, health facility delivery remained low at 44% and 42.6% in the early 1990s and in 2008 respectively [18,19]. Recent evidence on determinants of place of delivery in Kenyan utilising a nationally representative data and controlling for all factors is lacking, yet understanding the influences on place of delivery in Kenya is crucial to identifying key priority areas for policy and practise to increase the prevalence of skilled assisted deliveries.

We have used data from the 2008/2009 Kenya Demographic and Health Survey (KDHS) and linked them with a 2008 Kenyan Health Facility Database, that provides Global Positioning System (GPS) coordinates for distance analysis, to describe the factors that influence where women deliver in Kenya, and the reasons that women give for delivering at home.

## Methods

### Study population

The 2008/2009 KDHS is a nationally representative household-based survey, with interviewer administered questionnaires used to obtain a range of detailed health related and demographic information, and focussing on maternal and child health. Using the 1999 Kenya Population and Housing Census, a two-stage cluster sampling technique was used to sample 10000 households from 400 clusters and 8444 women aged 15–49 years and men age 15–54 years were interviewed. Details of the survey, sampling approach, including the questionnaires used, have been reported elsewhere [19]. In this study, after a description of all deliveries within the five years preceding the survey, we base the rest of the analysis on data for the most recent delivery for each mother.

The KDHS data collection procedures were approved by the ICF Macro (Calverton, Maryland), Institutional Review Board and the Scientific and Ethical Review Committee of the Kenya Medical Research Institute (KEMRI) and informed consent was obtained from respondents at the start of the individual interviews [19]. Permission to use these data was obtained from ‘Measure DHS’ [20]. No further ethical approval was necessary since the study was based on anonymous public use data with no identifiable information on survey respondents.

### Outcome and explanatory variables

Women were asked about “*place of delivery”* and whether this was *“at a health facility”, “at home”* or *“en route to a healthcare provider”*. The latter two responses were combined together for this analysis given that the latter group was small (1.14% (*n*=45)) to be analysed separately and reasoned that this may reflect women who attempt to deliver at home and only decide to go to a health facility much later. A subsidiary question asked for the *“main reason for home delivery”* with women selecting their main reason from the following list of ten options: facility too far/no transport, not necessary, abrupt delivery, cost too much, facility not open, don’t trust facility, not customary, family did not allow, no female provider, and other (unspecified).

From the questionnaire data available, we selected to analyse 16 explanatory variables which, based on a review of literature, have potential to influence place of delivery: maternal age, education, parity, marital status, number of ANC visits, healthcare provider at ANC, health facility of ANC, insurance, household size, relationship to household head, wealth index, presence of co-wife, rural/urban residence, ethnic group, region of residence and religion. These were classified for analysis under four broad themes: (1) socio-cultural factors, (2) perceived benefit/need of skilled attendance (3) physical accessibility, and (4) economic accessibility in a framework adapted by Gabrysch et al. (2009) from the Thaddeus and Maine’s three delays model (delay in decision to seek care, in reaching care and in receiving care) of delivery care use [21].

The wealth index, a proxy measure of a household’s long-term standard of living, is based on consumer goods, dwelling characteristics, type of drinking water source, toilet facilities, among others. Details of the philosophy and construction of the indices are discussed in detail by Measure DHS [22].

Maternal ages at delivery were computed from the mothers’ and babies’ birthdates. The distance of each household from the nearest health facility was calculated using GPS coordinates for households from the KDHS and for health facilities from the 2008 Kenya Health Facility Database obtained from Malaria Atlas Project (MAP) and developed by the Kenya Medical Research Institute (Kemri)-University of Oxford-Welcome Trust Collaborative Programme [23]. The Kenya Essential Package for Health as contained in The Second National Health Sector Strategic Plan of Kenya (NHSSP II), documents that all health facilities from level 2 dispensaries and clinics provided delivery services supervised by skilled health staff in 2004 [24] and therefore all health facilities contained in the health facility database are presumed to serve as a first point of contact in the healthcare system for a woman in labour. The household GPS coordinates were slightly displaced for each household after the survey to within 0-5 km in rural areas, 0-2 km in urban areas and 0-10 km in 1% of sparsely populated areas of Kenya to maintain confidentiality for respondents [25].

### Statistical methods

The bivariate associations between each potential risk factor and delivery at a health facility were explored, and those significant at p<0.05 were entered together into a multiple logistic regression model. Non-significant explanatory variables were removed from the model, and those excluded were re-entered in the model one at a time in a recursive process until all variables in the model were statistically significant and all excluded variables were not statistically significant, using the Wald test or Wald test for trend as appropriate. Pearson’s correlation matrix was used to check for collinearity between all variables and models fitted with and without adjustment for highly correlated variables.

To better understand the strongest effects, we explored associations between reasons given for home delivery and the factors that independently predicted place of delivery using cross-tabulation and chi-squared tests.

All analyses were conducted in Stata version 11.2

## Results

Of the 8444 women interviewed in the 2008/2009 KDHS, 3977 mothers had given birth to a total of 5857 babies within the preceding five years. Of these, 2493 (42.6%) took place at a health facility (public or private-sector health facility) and 3342 (57%) elsewhere. 21 (0.4%) deliveries did not have data on place of delivery.

3967 women provided data on where they delivered their most recent baby with 47% delivering in a health facility and a description of these women is given in Table 1. 98% (*n=*3878) of these women had complete data for inclusion in the final multivariate model. Nearly four fifths of these women lived in urban settings. The mother who lived closest to a health facility was 0.02 km away, while the farthest was 48.8 km away and 88% of all women lived within 5 km of a health facility.

**Table 1:** Descriptive data on the study population

**Variable**	**Frequency (%)**	**Variable**	**Frequency (%)**
***Place of delivery***	**3967**	***Perceived benefit/need***	
Home or on road	2109 (53.2%)	**No. of ANC visits**	**3892**
Health facility	1858 (46.8%)	None	290 (7.4%)
		1 visit	170 (4.4%)
***Socio-cultural factors***	2-3 visits	1560 (40.1%)	
**Maternal age**	**3967**	≥ 4	1872 (48.1%)
≤ 20 years	441 (11.1%)	**Birth order**	**3967**
21 – 34 years	2767 (69.8%)	1st delivery	845 (21.3%)
≥35years	759 (19.1%)	2^nd ^– 3^rd ^delivery	1531 (38.6%)
**Marital status**	**3967**	≥ 4^th ^delivery	1590 (40.1%)
Never Married	371 (9.4%)	**Prenatal Healthcare provider**	**3967**
Married	3035 (76.5%)	Doctor	882 (22.2%)
Living together	205 (5.2%)	Nurse	2723 (68.6)
Widowed	116 (2.9%)	TBA	74 (1.9%)
Divorced	34 (0.9%)	No ANC	288 (7.3%)
Living separately	206 (5.2%)	**Health facility of ANC**	**3959**
**Ethnic Group**	**3967**	Government Hospital	1026 (25.9%)
Kalenjin	610 (15.4%)	Health centre	956 (24.2%)
Kamba	432 (10.9%)	Dispensary	1050 (26.5%)
Kikuyu	628 (15.8%)	Private health facility	306 (7.7%)
Kisii	263 (6.6%)	Faith-based facility	266 (6.7%)
Luhya	617 (15.6%)	Other	67 (1.7%)
Luo	568 (14.3%)	No ANC	288 (7.3%)
Meru	194 (4.9%)	***Physical accessibility***	
Mijikenda/Swahili	215 (5.4%)	**Region**	**3967**
Somali	125 (3.2%)	Nairobi	268 (6.8%)
Other	314 (7.9%)	Central	370 (9.3%)
**Religion**	**3964**	Coast	330 (8.3%)
Roman Catholic	817 (20.6%)	Eastern	628 (15.8%)
Protestant/Christian	2696 (68.0%)	Nyanza	733 (18.5%)
Muslim	317 (8.0%)	Rift valley	1102 (27.8%)
No religion	119 (3.0%)	Western	440 (11.1%)
Other	15 (0.4%)	North Eastern	96 (2.4%)
**Household size**		**Residence**	**3967**
Median (range; IQR^3^)	5 ( 1 – 18; 4 – 7)	Urban	822 (20.7%)
**Presence of co-wife**	**3196**	Rural	3145 (79.3%)
No	2809 (87.9%)	**Distance**	**3956**
Yes	387 (12.1%)	<2 Km	1917 (48.5%)
**Relationship to household head**	**3967**	2 – 5 Km	1575 (39.8%)
Head	832 (21.0%)	>5 Km	464 (11.7%)
Wife	2373 (59.8%)	***Economic Accessibility***	
Other	761 (19.2%)	**Wealth index**	**3967**
**Maternal education**	**3966**	Poorest	841 (21.2%)
None	440 (11.1%)	Poor	762 (19.2%)
Primary	2483 (62.6%)	Middle	742 (18.7%)
Secondary	833 (21.0%)	Richer	762 (19.2%)
Higher	210 (5.3%)	Richest	860 (21.7%)
		**Insurance**	**3962**
		No	3744 (94.5%)
		Yes	218 (5.5%)

### Bivariate analysis

In bivariate analysis all explanatory variables were significant predictors of place of delivery (*p*≤0.001) (Table 2).

**Table 2:** **Unadjusted odds ratios for determinants of health facility delivery among women in Kenya**^**1**^

**Exposure variable**	**OR**^**2 **^**(95% CI)**	**P value**^**3**^	**Exposure variable**	**OR**^**2 **^**(95% CI)**	**P value**^**3**^
***Socio-cultural factors***			***Perceived benefit/need***		
**Maternal age**			**No. of ANC**^**2 **^**visits**		
≤20 years	1.00	<0.001*	≤1 visits	1.00	<0.001*
21 – 34 years	0.92 (0.69 – 1.22)		2 visits	2.68 (1.75 – 4.10)	
≥35 years	0.58 (0.41 – 0.81)		3 visits	4.05 (2.82 – 5.83)	
**Marital status**			≥ 4 visits	8.38 (6.01 – 11.7)	
Married	1.00	<0.001	**Birth order**		
Never Married	1.01 (0.75 – 1.37)		1^st ^delivery	1.00	<0.001*
Living together	1.10 (0.77 – 1.58)		2^nd^ delivery	0.53 (0.40 – 0.71)	
Widowed	0.56 (0.48 – 0.82)		3^rd^ delivery	0.30 (0.23 – 0.39)	
Divorced	0.85 (0.36 – 2.03)		≥4^th ^delivery	0.18 (0.13 – 0.26)	
Separated	1.23 (0.80 – 1.89)		**Prenatal healthcare provider**		
**Ethnic group**			Nurse	1.00	<0.001
Luhya	1.00	<0.001	Doctor	1.42 (1.05 – 1.91)	
Kalenjin	1.05 (0.57 – 1.91)		TBA^3^	0.22 (0.10 – 0.48)	
Kamba	1.34 (0.84 – 2.13)		No ANC	0.13 (0.07 – 0.24)	
Kikuyu	6.03 (3.45 – 10.52)		**Health facility of ANC**^**2**^		
Kisii	1.80 (1.16 – 2.80)		Govt hospital	1.00	<0.001
Luo	1.84 (1.25 – 2.72)		None	0.08 (0.04 – 0.14)	
Meru	5.19 (2.19 – 12.3)		Health centre	0.53 (0.39 – 0.72)	
Mijikenda/Swahili	1.30 (0.78 – 2.16)		Dispensary	0.34 (0.24 – 0.48)	
Somali	0.89 (0.45 – 1.79)		Private facility	2.15 (1.34 – 3.44)	
Other tribes	0.97 (0.52 – 1.81)		Faith-based	0.82 (0.46 – 1.45)	
**Religion**			Other	0.09 (0.03 – 0.29)	
Protestant/ Christian	1.00	<0.001	***Physical accessibility***		
Roman Catholic	1.01 (0.78 – 1.31)		**Region**		
Muslim	0.70 (0.43 – 1.14)		Nyanza	1.00	
No religion	0.20 (0.09 – 0.47)		Nairobi	10.7 (5.35 – 21.3)	<0.001
Other	4.56 (0.88 – 23.6)		Central	3.25 (2.10 – 5.01)	
**Household size**	0.42 (0.32 – 0.53)	<0.001	Coast	1.08 (0.73 – 1.59)	
**Presence of co-wife**			Eastern	1.10 (0.72 – 1.68)	
No	1.00		Rift Valley	0.57 (0.37 – 0.87)	
Yes	0.41 (0.30 – 0.55)	<0.001	Western	0.46 (0.31 – 0.69)	
**Relationship to household head**			North Eastern	0.25 (0.13 – 0.49)	
Head	1.00		**Residence**		
Wife	1.15 (0.93 – 1.421)	0.001	Rural	1.00	
Other	1.38 (1.08 – 1.76)		Urban	4.86 (3.39 – 7.07)	<0.001
**Maternal education**			**Distance from health facility**		
None	1.00		<2	1.00	
Primary	3.30 (2.10 – 5.19)	<0.001*	2 – 5 Km	0.50 (0.46 – 0.68)	<0.001*
Secondary	11.08 (6.63 – 18.5)		>5 Km	0.21 (0.13 – 0.35)	
Higher	42.0 (18.2 – 96.8)		***Economic accessibility***		
			**Wealth index**		
			Poorest	1.00	
			Poor	2.05 (1.44 – 2.91)	<0.001*
			Middle	3.55 (2.57 – 4.90)	
			Richer	5.30 (3.61 – 7.78)	
			Richest	18.3 (11.5 – 29.2)	
			**Insurance**		
			No	1.00	
			Yes	9.78 (6.47 – 14.79)	<0.001

* p values for trend; ^1^2008/09 Kenya Demographic and Health Survey Data; ^2^Unadjusted Odds Ratio ^3^Chi squared *p *values.

Mothers aged 35 or over, those married or widowed, with a co-wife and those living in larger households were less likely to deliver in a health facility. Place of delivery also differed between ethnic groups and religious groups with Muslim women and those without religion being less likely to deliver in a health facility than their Protestant/Other Christian counterparts. Having lower education reduced a mother’s likelihood of delivery in a health facility.

Those attending more antenatal care visits, and those with low parity were more likely to deliver in a health facility. The odds of health facility delivery also differed significantly depending on the healthcare provider during ANC and the health facility attended by the mother. The odds also differed significantly between the eight provinces of Kenya and mothers in rural areas, and those living further from a health facility, were less likely to deliver in a health facility.

Women from wealthier households and those having insurance cover were more likely to deliver in a health facility.

### Multivariate analysis

The socio-cultural factors that remained statistically significant in multivariate analysis were ethnic group and level of maternal education. When compared to Luhya women, Kalenjin, Kikuyu, Meru and Somali women were more likely to deliver in a health facility than other ethnic groups. Mothers with higher education were 7.46 times more likely to give birth in a health facility than those without any education (Table 3).

**Table 3:** **Adjusted odds ratios for determinants of health facility delivery among women in Kenya**^**1**^

**Exposure variable**	**Adjusted**^***2 ***^**OR (95% CI)**	**P value**
***Socio-cultural factors***		
**Ethnic group **(Luhya)	1.00	<0.001
Kalenjin	3.22 (1.51 – 6.87)	
Kamba	0.56 (0.27 – 1.17)	
Kikuyu	3.66 (1.72 – 7.74)	
Kisii	1.01 (0.48 – 2.09)	
Luo	1.36 (0.73 – 2.53)	
Meru	2.93 (1.14 – 7.52)	
Mijikenda/Swahili	1.16 (0.46 – 2.90)	
Somali	3.75 (1.15 – 12.31)	
Other	1.14 (0.54 – 2.40)	
**Maternal education **(None)	1.00	<0.001*
Primary	1.88 (1.11 – 3.18)	
Secondary	4.08 (2.24 – 7.46)	
Higher	7.46 (2.72 – 20.45)	
***Perceived benefit/need***		
**No. of ANC**^**3**^**visits** (≤1 visit)	1.00	<0.001*
2 visits	2.17 (1.14 – 4.13)	
3 visits	3.62 (2.02 – 6.48)	
≥4 visits	5.06 (2.88 – 8.90)	
**Birth order **(1^st ^delivery)	1.00	<0.001*
2^nd ^delivery	0.48 (0.35 – 0.67)	
3^rd ^delivery	0.33 (0.24 – 0.46)	
≥4^th ^delivery	0.35 (0.23 – 0.54)	
**Health facility of ANC**^**3 **^(Government Hospital)	1.00	<0.001
Health Centre	0.73 (0.54 – 0.98)	
Dispensary	0.52 (0.37 – 0.72)	
Private Health Facility	1.64 (1.00 – 2.69)	
Faith-based Facility	1.14 (0.65 – 1.99)	
Other	0.14 (0.04 – 0.56)	
***Physical accessibility***		
**Region **(Nyanza)	1.00	<0.001
Nairobi	2.18 (0.80 – 5.90)	
Central	1.11 (0.45 – 2.75)	
Coast	1.40 (0.62 – 3.15)	
Eastern	1.70 (0.82 – 3.54)	
Rift valley	0.36 (0.18 – 0.72)	
Western	0.59 (0.30 – 1.33)	
North Eastern	0.38 (0.12 – 1.21)	
***Economic accessibility***		
**Wealth index **(Poorest)	1.00	<0.001*
Poor	1.39 (0.94 – 2.06)	
Middle	1.96 (1.34 – 2.89)	
Richer	2.15 (1.42 – 3.27)	
Richest	5.62 (3.54 – 8.93)	
**Insurance** (No)	1.00	0.001
Yes	2.39 (1.33 – 4.31)	

^1^2008/2009 Kenya Demographic and Health Survey Data; ^2^Adjusted for the other variables in the table; ^3^Antenatal care; *p for trend;

Factors classified as relating to perceived need remained significant in multivariate analysis; those who did not attend, or attended fewer antenatal visits were less likely to deliver in a health facility, as were those whose ANC visits were in a health centre or dispensary when compared to those using government hospitals. Parity was also an independent predictor of place of delivery; women who had had four or more deliveries were 65% less likely to deliver in health facilities when compared to those for whom this was the first child.

Factors relating to economic accessibility were also significant in multivariate analysis; women from wealthier households and those with health insurance were more likely to deliver in a health facility.

The effect of region of residence remained significant in the multivariate model with women living in the Rift Valley province being less likely to deliver in health facilities when compared to women from Nyanza province while women from other regions did not differ significantly in place of delivery from those residing in Nyanza province after controlling for other factors. The effect of rural/urban residence or distance lived from a health facility were not statistically significant after controlling for other variables. However, there was a strong correlation between wealth and residence with residence becoming statistically significant in a model without wealth with women living in urban areas more than twice as likely to deliver in a health facility (Table 4).

**Table 4:** **Adjusted odds ratios for delivery in a health facility for residence without adjusting for wealth in Kenya**^**1**^

**Exposure variable**	**Adjusted**^**2 **^**OR, 95% CI**	**P value**
**Residence**		
Rural	1.00	<0.001
Urban	2.13 (1.47 – 3.10)	

^1^2008/2009 Kenya Demographic and Health Survey Data; ^2^Adjusted for ethnic group maternal education, Number of antenatal care (ANC) visits, place of ANC, birth order, region and insurance status;.

### Reasons for home deliveries

Of the 2115 women who delivered at home, 2103 (99.4%) provided their main reasons for not delivering in a health facility. Difficulty in physically accessing a health facility due to distance and/or lack of transport (36.9%) was the most common reason given, whilst 20.5% gave not being necessary as the reason (Table 5).

**Table 5:** **Association between distance and main reasons for not delivering at a health facility**^**1**^

		**Distance**^**2**^		***p*-value**^**3**^
	**Total (*n *= 2147)**	**<2 km (*n *= 777)**	**≥2 km (*n *= 1370)**	
Cost too much	237 (11.3%)	145 (14.6%)	92 (9.3%)	0.008
Facility too far/no transport	774 (36.9%)	361 (33.9%)	413 (38.8%)	
Not necessary	430 (20.5%)	211 (20.1%)	219 (20.8%)	
Abrupt delivery	382 (18.2%)	201 (19.4%)	180 (17.4%)	
Other^4^	275 (13.1%)	128 (12.0%)	146 (13.7%)	

^1^2008/2009 Kenya Demographic and Health Survey Data; ^2^ Straight distance of household from the nearest health facility; ^3^Chi-squared test *p *value ^4^Facility not open, don't trust facility, not customary , Family did not allow, no female provider and Others.

This pattern of the reasons given for home delivery did not significantly vary with rural/urban residence, level of education, parity or wealth but significantly differed with distance from health facility. Women living <2 km from a health facility were slightly more likely to say high costs and abrupt delivery were the main barriers to health facility delivery while those living ≥2 km away were more likely to give distance and/or lack of transport as the main reason (Table 5). Nevertheless, almost 60% of those living ≥2 km away gave reasons other than distance as their main reason for not delivering in a health facility.

## Discussion

### Summary of results

In Kenya, about 53% of deliveries take place outside health facilities despite more than 88% of mothers living less than five kilometres from a health facility and 93% of pregnant women having at least one ANC visit during pregnancy. Higher levels of education, low parity, optimally attending ANC services and having insurance cover increase the likelihood of delivering in a health facility. Place of delivery also varies significantly among different ethnic groups, regions of residence, and with the type of health facilities mothers use. Distance from a health facility did not significantly predict place of delivery.

Difficulty in physically accessing health facilities was the most reported reason for not delivering in a health facility but about 60% gave a reason other than distance and/or lack of transport including 20.5% of mothers who reasoned that delivering in a health facility was not necessary. This pattern was regardless of wealth, parity, education or rural/urban residence. Women living near a health facility were slightly more likely to report delivering at home due to abrupt delivery and high costs.

### Strengths and limitations of study

This study uses nationally representative data and multivariate methods to identify independent risk factors for place of delivery in Kenya. The main strength of this study is its linkage of household and health facility GPS data to compute and control for improved estimates of distance of households from a health facility. Given that with current coverage of health facilities, distance does not significantly determine place of delivery, this paper suggests the need to also focus on other barriers to access to health facilities as a strategy to improve health facility delivery. As a study based on data from a single country, the confounding effect of wider contextual factors such as the healthcare system is removed giving better estimates of the effect of other variables.

The study has some limitations. Data on child birth and place of delivery were collected retrospectively from mothers so there is potential for recall bias; to minimise this, we have used data on the most recent birth within five years of the KDHS. Data on exposure variables such as wealth, distance and residence reflected the situation at the time of the survey and not at the time of delivery and hence mothers may have shifted from one category of classification into another. Such non-differential misclassification may have reduced the strengths of observed associations. The distance used in this study is an estimate of the true distance given the straight line distance used. We also have no data on the quality of the roads, and we have not looked at whether there are seasonal changes in the impact of distance, such as might arise from seasonal variations in road quality, due to lack of necessary data to this effect. The displacements in household GPS coordinates introduce some misclassification of computed distance and the differential displacement measurements in urban and rural areas means there is more inaccuracy in the rural measurements. However there was only weak correlation between distance and rural/urban residence (Pearson’s r 0.35) and none of them was statistically significant with the exclusion of the other in the final model.

There was no appropriate information on several other important determinants of maternal health service use during childbirth including the occurrence of emergencies during home deliveries that prompt seeking professional assistance and outcomes from past healthcare service use, and hence these could not be controlled for. The outcome for this study, mothers who delivered their most recent baby in a health facility, is a common event (prevalence of 47%) therefore the rare disease assumption does not hold, and odds ratios therefore overestimate the relative risk of delivering in a health facility. There were 177 households with two mothers and 11 households with 3 mothers but since the clusters were very small (≤3) and affected a small percentage of the dataset (9%), the impact on our findings is expected to be minimal [26]. Lastly, this study relied solely on quantitative data, and it is important that a better understanding of the effects of specific social-cultural factors that might underlie the effect of variables such as ethnicity on place of delivery are explored through future qualitative study.

### Results set in context

Findings on the factors that determine where women deliver in Kenya are comparable to those from other developing countries where education, ANC utilisation, parity, residence, ethnicity and wealth have been shown to be important factors [7,9-11,15]. Ochako et al. have also shown that in Kenya, similar factors determined the timing of the first ANC and the type of assistance young mothers aged between 15–24 used during childbirth, with a strong association between early seeking of ANC and skilled assistance during childbirth [14].

In this study, despite physical access being the most cited reason for home delivery, it is interesting that the effect of distance on place of delivery was not statistically significant after controlling for other factors. Given the proportion of mothers attending at least one ANC visit during pregnancy, and the proportion of women living within 5 km of a health facility, it is reasonable to argue that health facilities are within physical reach. Inability to access appropriate means of transport during labour may explain a significant proportion of mothers unable to physically access health facilities during childbirth. Increasing facility numbers for delivery in Kenya, while important, may not necessarily improve physical accessibility without provision and promotion of appropriate and affordable means of transport for mothers in labour. A systematic review has showed that better community referral/transport systems have increased rates of skilled attendance at birth in other contexts [27].

Having insurance doubled the odds of a health facility delivery suggesting that inability to pay for services is an important barrier, but when asked, only a small proportion of mothers cited high health facility costs as their main reason for delivering at home. The strong collinearity between wealth status and rural/urban residence suggests that economic factors are important for rural mothers for whom there was considerable inequality in place of delivery. Collectively, this evidence may be indicative of the fact that the effect of poverty on where a mother delivers is not solely through inability to pay for services. Poverty may indirectly increase the odds of home delivery by raising the opportunity cost of delivering at a health facility, making delivery at home a rational cheaper option especially in rural areas where health facilities are more likely to be distant. Paying for transport, costs of time for the mother and family members while at the hospital, and the need to continue taking care of the rest of the family and the home become important considerations especially in the context of low perception of need for health facility delivery. Studies in different contexts have found that time costs, travel costs, direct payments, and fear of unofficial payments can be barriers to the use of maternity services [28-30].

We note a decreasing trend in health facility delivery with increasing parity and *“not being necessary”'* as the second most cited reason for home deliveries. Theoretical models of healthcare utilisation suggest that outcomes from health facility use and perception of need significantly determine where women deliver [31] with previous evidence supporting the significance of these factors [7]. A study in rural parts of Tanzania found that staff attitudes and poor treatment including lack of privacy at health facilities discouraged women from delivering there [15] while in rural parts of Nigeria women cited unsatisfactory services as their main reasons for home delivery [16]. The failure to perceive the need to deliver subsequent children in health facilities may arise from experiences and outcomes of previous utilisation of health facilities during childbirth which subsequently inform the subjective valuation of the need. This may also explain the differences in place of delivery among women using different levels of health facilities in the Kenyan healthcare system. A study in Nyanza province in Kenya found that lower level facilities were more likely to provide poor quality maternal services than higher level health facilities [32] and hence mothers using such facilities may be more likely to believe health facility delivery to be unnecessary. While health education around pregnancy and childbirth may improve knowledge, perception and valuation of health facility delivery, this will need to be accompanied by improvement of the experience, responsiveness and care for women during delivery at health facilities alongside addressing other barriers if this is to translate into seeking and using health facilities for childbirth.

Although marital status, maternal age, relationship to household head and religion have been found to determine place of delivery in other contexts [10] and Stephenson *et al*. also found marital status, maternal age and religion to be important determinants of place of delivery in Kenya [9], these did not independently predict place of delivery in this study and that did not change even when modelled without controlling for ethnic group and region of residence.

## Conclusions

Physical access to health facilities due to lack of access to timely and appropriate transport, and economic considerations, are important barriers for women to deliver at health facilities in Kenya. Many women do not perceive a need to seek health facility delivery and increasingly deliver their subsequent children at home. We conclude that subjective valuation of the need deteriorates with subsequent births.

There are several implications of our findings on strategies to promote skilled assisted deliveries in Kenya. Improving physical access by facilitating access to appropriate and affordable transport during labour, and improving the experiences and outcomes of mothers seeking health facility delivery may increase its uptake. This should be augmented by health education interventions that improve the attitudes and subjective value placed on health facility delivery by pregnant mothers, lowering its opportunity cost and hence increasing demand. Mechanisms to ensure services are affordable at point of service delivery will be an important adjuvant to this strategy.

## Abbreviations

ANC: Antenatal care; GPS: Global positioning system; KDHS: Kenya demographic and health survey; KEMRI: Kenya medical research institute; MAP: Malaria atlas project.

## Competing interests

The authors declare that they have no competing interests.

## Authors' contributions

JK: Participated in the origination of the study and its design. He also conducted the literature review, data analysis, writing the results and discussion sections. SL: Participated in the conceptualisation and design of the research idea, data analysis and writing the results and discussion sections. GD: Participated in refining the initial research idea, writing the background section and critically reviewed the drafts. All authors read and approved the final manuscript.

## Pre-publication history

The pre-publication history for this paper can be accessed here:

http://www.biomedcentral.com/1471-2393/13/40/prepub
